# Personality Traits of Croatian University Students with Internet Addiction

**DOI:** 10.3390/bs12060173

**Published:** 2022-06-01

**Authors:** Ivan Miskulin, Ivana Simic, Nika Pavlovic, Jelena Kovacevic, Ivica Fotez, Goran Kondza, Hrvoje Palenkic, Vesna Bilic-Kirin, Marinela Kristic, Maja Miskulin

**Affiliations:** 1 Faculty of Medicine Osijek, Josip Juraj Strossmayer University of Osijek, 31000 Osijek, Croatia; ivana.simic.osijek@gmail.com (I.S.); nika.pavlovic@mefos.hr (N.P.); dr.kovacevic.jelena@gmail.com (J.K.); gkondza@gmail.com (G.K.); vesna.bilic.kirin@gmail.com (V.B.-K.); 2 Faculty of Dental Medicine and Health Osijek, Josip Juraj Strossmayer University of Osijek, 31000 Osijek, Croatia; ivica.fotez@vt.t-com.hr (I.F.); hrpal@net.hr (H.P.); 3 Department of Psychiatry, University Hospital Split, 21000 Split, Croatia; kristicmara@gmail.com

**Keywords:** Internet, addictive behavior, university, students, personality inventory, Croatia

## Abstract

Specific personality traits may predispose individuals to various forms of addictive behaviors. This study aimed to investigate the association between personality traits of university students and Internet addiction (IA). A sample of 1051 university students was recruited from the largest university in Eastern Croatia. A structured anonymous questionnaire that included questions regarding students’ sociodemographic information and Internet usage patterns, the Young Internet Addiction Test and Big Five Inventory served as a research tool. The study revealed that 1.0% of the studied sample expressed severe IA while 24.6% of study participants expressed some signs of addiction. The IA was detected in 576 (80.0%) students who used the Internet mainly for social networking, in 30 (78.9%) students who mainly used it for online gaming, and in 153 (52.2%) students who mainly used it for university assignments (*p* < 0.001). Higher neuroticism, higher extraversion, and higher openness to new experiences were connected with IA in general (*p* < 0.001). Higher neuroticism, higher extraversion, and higher openness to new experiences were significantly associated with addictive behavior during social networking (*p* < 0.001). Higher extraversion and higher openness to new experiences were significantly associated with addictive behavior during Internet usage for university assignments (*p* = 0.025), while there were no significant associations between specific personality traits and addictive behavior during online gaming (*p* = 0.059). Personality traits must be taken into account while developing programs and implementing interventions for preventing IA in the university student population.

## 1. Introduction

Society has been hugely impacted by the Internet since it is highly accessible and able to pass on information, enable contact with people, and exchange content [1]. These characteristics of the Internet can improve people’s quality of life but also present a risk when without control [2,3]. Internet users are increasing in number all over the world [1,3]. Healthy Internet use provides individuals a chance to employ various cognitive and social skills, but overuse of the Internet may induce a syndrome that affects one’s social, psychological, and professional life [4]. Internet addiction (IA) presents a new type of non-chemical addiction, characterized by one’s inability to inhibit Internet use despite negative effects on many domains of life, such as academic performance, social relations, physical and mental health, and quality of life [1,3,4,5,6]. As the media dependency theory [7] implies, Internet addiction is a part of the audience-media-society relationship, and its probability is higher for those persons with higher reliance on media for various uses and gratifications, as well in times of rapid social change where alternative sources of reliable information are not readily available. Thus, it can also be concluded that its importance and dangers will be more pronounced in times of the increasing importance of online connections [8,9].

Even though IA has attracted lots of attention from researchers all over the world [10], there are still some knowledge gaps concerning this issue that need to be fulfilled. Namely, to have a clear global picture of IA, there is a need for data regarding the pattern and correlates of IA among young adults, including the university and college students from different regions and countries of the world. Research has underlined that various cultures psychologically differ in a variety of personal characteristics, for example, cognition, personality traits, and emotions, and because of that, the nationally differing effects of the aforementioned characteristics on the development of the IA can be expected [11]. Following the latter, as well as the fact that there is a dearth of such studies in Croatia, the present study focused on IA characteristics among Croatian university students. The university or college students are at risk of developing IA due to several important reasons [12,13,14]. They have several advantages regarding Internet availability: comparatively easier and wider access via campus library, free campus wireless Internet zones, and cheaper mobile Internet packages for students. Furthermore, these young adults, irrespective of their living arrangements, whether they are living with their family or in the university dormitories, usually experience the freedom of life choices, including the use of the Internet. Their development stage is characterized by searching for and determining their identity, partner and career, and they tend to use the Internet to achieve all of those things, exposing themselves to a risk of excessive Internet use and misuse over time [12,14].

Personality represents the fundamental characteristics that affect human behavior [15]. It has been consistently found that personality traits are associated with IA in different populations and cultures [6,14,15,16,17,18]. One of the most prominent personality models is the Five-Factor Model of personality [19]. According to this model, personality can be described based on five major factors (commonly referred to as the Big Five), and these are extraversion, agreeableness, conscientiousness, neuroticism, and openness to experiences [15,19,20].

This study aimed to investigate the association between personality traits of Croatian university students and IA.

## 2. Materials and Methods

### 2.1. Participants

This cross-sectional study was conducted from 1 January to 28 February 2020 among 1.051 students from the University of Osijek in Eastern Croatia. The study was approved by the Ethics Committee of the Faculty of Medicine Osijek, Croatia (Ethical Approval Code: 2158-61-07-20-12).

The University of Osijek is the largest in Eastern Croatia, with 16.065 students, 40.7% of males and 59.3% of females. A total number of 1.500 questionnaires were delivered randomly to second-year undergraduate students and first-year graduate students from all faculties within the University of Osijek. The second-year undergraduate students and first-year graduate students were chosen to be the study participants to explore the significance of study duration for the prevalence of IA among university students who were studying for two or three years and those who were studying for four or more years. The overall response rate was 70.7% (1.060/1.500). Out of a total of 1.060 completed questionnaires, 9 were excluded from further statistical analyses since they were incomplete. The final sample size of 1.051 study participants presented 6.5% of all students studying at the University of Osijek and was a representative cross-faculty sample. The students were invited to participate in the study voluntarily and were approached in lecture theaters immediately after the completion of a lecture. The researchers explained the study and provided the questionnaire to those willing to participate who signed informed consent. It took about 15 min to fill out the entire questionnaire, and then study participants were instructed to put these filled-out forms in a specially designed box that was positioned by the door of the lecture theater and could not be opened or seen through.

### 2.2. Measures

A structured anonymous questionnaire comprised of three parts served as a research tool. The first part of the study questionnaire served to collect sociodemographic information about students, such as their age, sex, year of study, repetition of the year of study, faculty subject area, type of housing during the study, and status of employment while studying as well as the data on the main reason for the usage of the Internet.

The second part of the study questionnaire included the Young Internet Addiction Test (IAT). The IAT is the most widely used measure for IA worldwide [1,6,16,21]. The IAT, a validated and reliable measure of addictive use of the Internet, is a 20-item self-rated questionnaire that measures the severity of Internet addiction [21,22]. The IAT has been validated in Croatia, where on the sample of high school students aged 15–20 years, high internal consistency of the whole test was determined (internal consistency coefficient, i.e., Cronbach alpha on this sample was 0.91) [23]. Each item is scored on a 6-point Likert scale, from 0 (“never”) to 5 (“always”), with a total score ranging from 0 to 100. The test measures the extent of involvement with the Internet and classifies four possible patterns of Internet usage as follows: a normal level of Internet usage or absence of addiction (total score ranging from 0 to 19); a low level of IA or average Internet user (total score ranging from 20 to 39); a moderate level of IA or problematic Internet user (total score ranging from 40 to 69) and severe level of IA (total score ranging from 70 to 100) [22,23,24]. A score in the low or moderate range only suggests possible IA, but it does not define it [21].

The third part of the study questionnaire included the short, 10-item version of the Big Five Inventory (BFI-10) [25]. The BFI-10 assesses personality traits according to the Five-Factor Model of personality based on five major factors: extraversion, agreeableness, conscientiousness, neuroticism, and openness to experiences [10,17,25,26]. The BFI-10 comprises 10 items on a 5-point Likert scale from 1 (“strongly disagree”) to 5 (“strongly agree”), with two items for each personality dimension [17,26]. Consequently, the minimum and maximum scores of each dimension are 2 and 10, respectively. The highest scored personality dimension is identified as the dominant personality trait of the participant [10]. The psychometric properties and the validity of BFI-10 have been demonstrated as being appropriate [25,26,27].

### 2.3. Data Analysis

Statistical analyses were conducted using the IBM SPSS Statistical Package, version 22.0 (SPSS Inc., Chicago, IL, USA). The normality of the data distribution was confirmed by the Kolmogorov–Smirnov test, whereupon all data were processed by the methods of descriptive statistics. The categorical variables were described in absolute and relative frequencies, while the numerical variables were described as median and interquartile ranges. The χ2-test and Fisher exact test were used for the comparison of categorical variables between the groups. In all statistical analyses, two-sided *p*-values of 0.05 were considered significant. 

## 3. Results

The characteristics of all study participants are shown in Table 1. The median age of all study participants was 22.0 years, with an interquartile range from 20.0 to 23.0 years. The median age of the second-year undergraduate students was 20.0 years with an interquartile range from 20.0 to 21.0 years, while the median age of the first-year graduate students was 23.0 years with the interquartile range from 22.0 to 24.0 years.

Considering the main reason for the usage of the Internet among all study participants, there were 293 (27.9%) students who used the Internet mainly for the fulfillment of their university assignments, 720 (68.5%) students who used it mainly for social networking, and 38 (3.6%) students who used it dominantly for online gaming.

**Figure 1:**
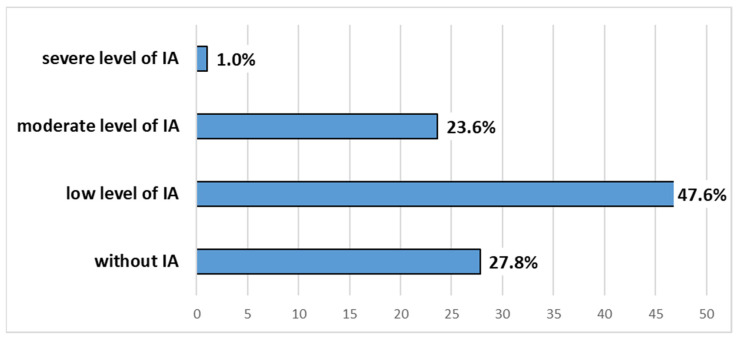
All study participants according to the IAT total score.

All study participants, according to the IAT total score, were divided into four categories that are shown in Figure 1. Following the IAT total score, it can be said that there were 292 (27.8%) study participants without IA (IAT total score between 0 and 19) and 759 (72.2%) study participants with some level of IA (IAT total score between 20 and 100).

The IA was more frequent among males (χ2-test; *p* = 0.024), among students who had repeated the year of study (χ2-test; *p* = 0.012), and among younger students (χ2-test; *p* = 0.027). There were no statistically significant differences in IA regarding the participants’ year of study (χ2-test; *p* = 0.082), the participants’ faculty subject area (χ2-test; *p* = 0.134), the participants’ type of housing during the study (χ2-test; *p* = 0.666), and the participants’ status of employment while studying (χ2-test; *p* = 0.138) (Table 2). There was a statistically significant difference in IA according to the main reason for the usage of the Internet (χ2-test; *p* < 0.001) (Table 3).

Considering the interconnection between personality traits and IA, the study revealed that higher neuroticism, higher extraversion, and higher openness to new experiences were significantly connected with IA in general (χ2-test; *p* < 0.001). Higher neuroticism, higher extraversion, and higher openness to new experiences were significantly associated with addictive behavior during social networking (χ2-test; *p* < 0.001). Higher extraversion and higher openness to new experiences were significantly associated with addictive behavior during Internet usage for university assignments (χ2-test; *p* = 0.025), while there were no significant associations between specific personality traits and addictive behavior during online gaming (χ2-test; *p* = 0.059).

Finally, the study revealed that the riskiest personality trait for the IA in the case of university assignments as the main reason for Internet usage was consciousness, and the riskiest personality trait for the IA in the case of social networking as the main reason for Internet usage was neuroticism, and the riskiest personality trait for the IA in the case of online gaming as the main reason for Internet usage was openness to new experiences (Fisher exact test; *p* = 0.039) (Table 4).

## 4. Discussion

This study revealed that IA is a serious public health issue among the university student population in Croatia, whereas 1.0% of the studied sample expressed severe IA according to the IAT total score (with a cut-off point of 70). The discovered prevalence of severe IA in Croatian university students is comparable but somewhat different from the prevalence of severe IA in the university student population in some other studies that employed the same research tool with the same cut-off value. For example, the study among Mexican university students [28] revealed the prevalence of severe IA to be 0.2, while the study among Japanese university students discovered that the prevalence of severe IA was 3.3 [29]. The meta-analysis study from 31 countries that deployed IAT as a research tool with the same cut-off value as the one used in our study showed that the global prevalence of IA was around 6.0%, with the highest prevalence found in the Middle East at 10.9%, which followed North America at 8.0%, Asian countries with around 7.1%, Southern and Eastern Europe at 6.1%, Oceania at 4.3% and finally the lowest prevalence was found in Northern and Western Europe at 2.6% [30]. The cross-sectional study that included college and university students in eight countries and used the Generalized Problematic Internet Use Scale-2 with the cut-off value of 65 that was published in 2019 revealed the following prevalence of IA: Croatia at 2.5%, Serbia at 1.6%, Turkey at 4.8%, India at 12.2%, UAE at 12.1%, Nepal at 12.6%, Bangladesh at 10.8%, and Vietnam at 11.7%. The aforementioned study showed a similar pattern of IA across the world regions such as the one detected by the meta-analysis published in 2014 [30], and the prevalence of IA detected in Croatia is comparable, although somewhat higher than the one that we found in our research [12]. The particularly troublesome fact that was discovered in this study was the fact that 23.6% of study participants fell into the category of moderate IA, meaning that 24.6% of study participants were showing some signs of addiction, suggesting the risk of more serious problems in Internet usage.

Considering the sex difference in IA, this study showed that IA was statistically more frequent among males than among females, which is consistent with several studies from different countries [17,28,31,32,33] as well as with the results of previous studies in Croatia [34,35] although some studies found that the IA is more frequent among females [26,36] and others did not discover the difference in IA considering sex [6,12,29,37].

The present study further revealed that IA was more frequent among students who repeated the year of the study, which points to the fact that, indeed, IA is connected with a lower academic performance, which is in line with the findings of some similar studies [36,38,39,40] although some studies did not find the connection between IA and students’ academic performance [3,41].

This study did not find the difference in IA prevalence regarding the participants’ year of study, pointing to the fact that the duration of student life itself was not a risk factor for IA, but when analyzing the prevalence of IA by students’ age groups, then this study proved that younger age of students puts them into higher risk for IA development, which is consistent with other similar studies [42,43,44] and contrary to the results of recent international study in eight countries that did not find an association between age and problematic internet use [12].

Our study did not find a connection between the type of housing during the study and IA that points to the fact that in the Croatian university student sample, parental control of Internet usage as a protective factor in IA development was not significant, although some other studies showed that IA was negatively related to parental control [45,46].

The present study did not find a difference in the IA prevalence considering the participants’ faculty subject area, which is in concordance with an earlier study among Croatian university students [35] and a recent study among Indian students [33], although a recent study from Egypt found that students who studied in the field of biomedicine a more prone to IA development [47] and study among Indian students published in 2020 showed that students who studied in the field of technical sciences are the most vulnerable for the development of IA [48].

Finally, this study did not discover the difference in the IA prevalence regarding the employment status of students while studying. National Eurostudent report for Croatia on social and economic conditions of student life in Croatia has confirmed that students who work during their study in Croatia are more often from families of lower socioeconomic status [49]. When we look at our results regarding the employment status of students while studying and IA from that angle, we can say that our study did not find a connection between students’ socioeconomic status and development of IA, which complies with the results of other similar studies [50,51] although some researchers found that there is a negative trend between socioeconomic status and the prevalence of IA meaning that lower socioeconomic status is connected with higher prevalence of IA [52].

The study showed that Croatian university students used the Internet mainly for social networking, which was shown in previous studies among university students in Croatia [34,35] and is in concordance with some other similar studies [53,54]. When looking at the prevalence of IA in connection to the main reason for Internet usage, our study showed that Croatian university students who mainly use the Internet for social networking and online gaming had a significantly higher prevalence of IA in comparison to those students who mainly used Internet for the fulfillment of their university assignments, which is in concordance with the results of other similar studies who had all proved that social networking and online gaming are posing a greater risk for IA development than the usage of Internet for job or university assignments [12,36,53,55,56,57]. A recent international study in eight countries showed that Croatia was one of the countries, along with the UAE, Turkey, and Vietnam, in which the Internet usage mainly for social networking was significantly associated with IA [12], which was also revealed in two previous studies in Croatian university students [34,35]. However, this study demonstrated another important issue regarding IA in the university student population, and that is that 52.2% of students who mainly used the Internet for the fulfillment of their university assignments were considered addicted to the Internet following the used criteria meaning that the use of the Internet for required work is not easily separated from the use of the Internet because of an addiction. The described problem of the addiction versus reliance on Internet usage for required work in the university student population has been previously identified in the literature [58], and it is important to emphasize that the results of this study should be seen in the light of that problem, so that “addiction” among university students may not be as clear-cut as one might initially believe.

The present study confirmed that personality traits had a meaningful association with IA, which was also found in several other studies [10,15,18,59,60,61] and further revealed that Croatian university students’ higher neuroticism, higher extraversion, and higher openness to new experiences were significantly connected with IA. This finding is in concordance with the results of other similar studies that showed how higher neuroticism is positively related to IA, meaning that neuroticism can be a risk factor for IA development [6,14,15,18,26,55,61,62,63,64]. The explanation for the observed connection lies in the fact that individuals with higher neuroticism experience more troubled relationships and distressed situations, and because of that, they are more prone to use the Internet to avoid the aforementioned unpleasant experiences [18]. The results of this study also showed that there was a significantly positive association between higher extraversion and IA, and these findings are consistent with the findings of other similar studies [18,65,66]. These findings can be explained by the core characteristics of extraversion that is somewhat dual. Namely, extraversion is related to how individuals interact with others, and it is known that extroverted individuals usually have suitable interpersonal relationships and adequate social support in life, and because of that, they do not have a need to seek more friends and social support online. On the other hand, extroverted individuals are, at the same time, often impulsive and tend to search for new stimulation, which makes them more prone to engage in addictive behavior [18]. However, contrary to our results, some studies found that extraversion was negatively associated with IA [15,16,61,62,63,67,68,69]. Considering the openness to new experiences, our study revealed that there is a positive association between higher openness to new experiences and IA, which had also been shown in other similar studies [16,18,69]. The explanation for the observed association between openness to new experiences and IA lies again in the dual nature of this personality dimension. Individuals with high openness to new experiences often have a wide range of interests and activities, and because of that, they spend less time online. Simultaneously with the latter, the individuals who are highly open to new experiences are also imaginative, artistic, curious, open-minded, and inclined to new stimulations and activities, thus having an increased risk for the IA development [18]. However, there are some studies that, contrary to our results, found how openness to new experiences was negatively correlated with IA [15,68].

Considering the association of IA developed during social networking and personality traits, like in this study, some other studies also found that higher neuroticism is positively correlated with addictive behavior during social networking [17,20,70,71,72], proving that neurotic individuals are more prone to excessive Internet usage for social purposes. Considering the extraversion, this study showed that higher extraversion was significantly associated with addictive behavior during social networking, which complies with the results of some similar studies [17,19,73]. However, opposite the latter, Kircaburun and Griffiths, in their study on university students in Turkey, found that extraversion and neuroticism were not related to addictive behavior during social networking [10]. This study finally showed that higher openness to new experiences in an observed sample of Croatian university students was significantly associated with addictive behavior during social networking, which is opposite to the results of a study performed by Blachnio et al. (2017) who found that being less open to new experience is a risk factor for IA development during social networking [67]. When comparing the personality traits that were connected with the IA in general and IA during social networking, our study proved that even though the IA, in general, is different from addictive behavior during social networking, online social networking addiction has the biggest overlap with IA more generally in the sense of personality traits influence.

The very interesting and highly important novel finding of this study was that some personality traits such as higher extraversion and higher openness to new experiences were significantly associated with addictive behavior during Internet usage for university assignments, which, to the best of our knowledge, had not been investigated and proven elsewhere.

Regarding the association between personality traits and addictive behavior during online gaming, this study did not show significant associations between specific personality traits and addictive behavior during online gaming, which is opposite to the results of other similar studies. Following that, Wang et al. (2012) found that openness to new experiences was positively related to playing online games and that there was a negative relationship between extraversion and playing online games, while Wang et al. (2015) found that low openness to new experiences and low conscientiousness were significantly associated with gaming addiction [17,20]. In their study, Müller et al. (2014), however, found that addictive behavior during online gaming was associated with higher neuroticism, decreased conscientiousness, and low extraversion [74]. Finally, Saini et al. (2016), similarly to latter research, found that addictive behavior during online gaming was positively correlated with neuroticism and negatively correlated with extraversion [75].

Lastly, when we directly analyzed the influences of Internet usage patterns on Internet addiction and its relation to personality traits present study revealed that specific personality traits were riskier for the IA in the case of different online activities, i.e., it was shown that the riskiest personality trait for the IA in the case of university assignments as the main reason for Internet usage was consciousness, the riskiest personality trait for the IA in the case of social networking as the main reason for Internet usage was neuroticism, while the riskiest personality trait for the IA in the case of online gaming as the main reason for Internet usage was openness to new experiences. The latter discoveries are highly important novel findings that emphasize the role of Internet usage patterns and their associations with specific personality traits as an important part of IA development etiology in an observed highly vulnerable population of university students.

The results of our study should be interpreted by taking into account the limitations and strengths of the present study. First, due to the cross-sectional design of our study, only associations and not a causal link between investigated variables could be established. Second, the usage of self-report-based instruments for assessing the patterns of Internet usage and severity of IA as well as the features of individual’s personality traits was prone to social biases such as social desirability of collected answers. Third, the voluntary participation of students in our study might introduce potential response biases to our research.

Besides the limitations, our study also poses several important strengths. First, the study was based on a large and representative cross-faculty sample of the biggest university in Eastern Croatia, thus allowing us to easily generalize our data to the whole Croatian student population. Second, bearing in mind that to the best of our knowledge, this was the first study that explored associations between an individual’s personality traits and patterns of Internet usage and characteristics of IA in a vulnerable population of university students in Croatia and taking into account how personality traits are strongly influenced by specific cultural peculiarities of different countries allow us to state that the significance of this study goes beyond the national framework because many studies in different countries are needed to fully understand complex interconnections between individual’s personality traits and previously mentioned Internet-related variables. Third, the study employed validated instruments that had been previously used in other similar studies allowing us to easily compare our results with the significant research in the subject area and to draw very important evidence-based conclusions as a starting point for appropriate preventative interventions.

The present study has raised some new questions that should be answered in future research. The first of them is whether individuals are addicted to the Internet in general or to certain activities on the Internet. Accordingly, future studies should separately investigate the IA connected with social networking with a distinction between different social media that are highly popular among youth, such as Instagram, Twitter, etc., and also separately investigate the IA connected with online gaming. All of these studies should be specifically controlled for work-related online activities to avoid possible biases if reliance on Internet usage for required work is unintentionally perceived as addiction. Bearing in mind that the importance and dangers of IA will be more pronounced in times of the increasing importance of online connections, such as the global pandemic of COVID-19, these influences should certainly be taken into account in any future study. Finally, the causal relationship of investigated variables can be established only in longitudinal studies; thus, this study design should be employed in the future investigation of IA and its correlates in vulnerable populations.

Nevertheless, the findings of this study have significantly contributed to a better understanding of IA in the university student population. The practical implications of this study’s findings are reflected in the possibilities for planning interventions with university students in the field of problematic Internet usage. The results of this study are therefore useful to practitioners working with children and youth, especially specialists in school medicine, and besides that, they are also the source of new and valuable insights for the international scientific community.

## 5. Conclusions

Internet addiction proved to be one of the crucial public health issues in the Croatian university student population, and the results of this study significantly contributed to the understanding of the etiology of IA in this highly vulnerable young population. The study emphasizes the important role of individual personality traits in IA development among the university student population. Knowledge regarding the complex associations of university students’ personality traits and the development of IA in university students can open the possibilities for the development of different models of preventative public health interventions in the observed population. Hence, the findings of our study may help to reduce the adverse effects of IA by making sure that preventative interventions are sensitive to university students with different personality traits.

## Figures and Tables

**Table 1:** Characteristics of all study participants.

Study Participants Characteristics	N	%
Sex		
Male	414	39.4
Female	637	60.6
**The year of study**		
Second-year undergraduate students	597	56.8
First-year graduate students	454	43.2
**Age groups**		
Younger (students aged 19 to 21 years)	516	49.1
Older (students aged 22 or more years)	535	50.9
The faculty subject area		
The social field of science	364	34.6
The technical field of science	80	7.6
The biotechnical field of science	243	23.1
The field of biomedicine and natural field of science	214	20.4
The field of humanities and arts field	150	14.3
**The repetition of the year of study**		
No	863	82.1
Yes	188	17.9
**The type of housing during the study**		
Living with family or relatives	373	35.5
Living in the dormitory or rented flat	678	64.5
**The status of employment while studying**		
Working while studying	411	39.1
Not working while studying	640	60.9

N—number of participants.

**Table 2:** Participants with or without IA according to their sociodemographic characteristics.

Participants Sociodemographic Characteristics	N (%)			*p* *
	Participants with IA	Participants without IA	Overall	
**Sex**				
Male	315 (76.1)	99 (23.9)	414 (100.0)	0.024
Female	444 (69.7)	193 (30.3)	637 (100.0)	
**The year of study**				
Second-year undergraduate students	444 (74.4)	153 (25.6)	597 (100.0)	0.082
First-year graduate students	315 (69.4)	139 (30.6)	454 (100.0)	
**Age group**				
Younger (19–21 years)	389 (75.4)	127 (24.6)	516 (100.0)	0.027
Older (22 or more years)	370 (69.2)	165 (30.8)	535 (100.0)	
**The faculty subject area**				
The social field of science	266 (73.1)	98 (26.9)	364 (100.0)	0.134
The technical field of science	54 (67.5)	26 (32.5)	80 (100.0)	
The biotechnical field of science	189 (77.8)	54 (22.2)	243 (100.0)	
The biomedicine and natural sciences	147 (68.7)	67 (31.3)	214 (100.0)	
The field of humanities and arts field	103 (68.7)	47 (31.3)	150 (100.0)	
**The repetition of the year of study**				
Yes	150 (79.8)	38 (20.2)	188 (100.0)	0.012
No	609 (70.6)	254 (29.4)	863 (100.0)	
**Type of housing during the study**				
Living with family or relatives	266 (71.3)	107 (28.7)	373 (100.0)	0.666
Living in the dormitory or rented flat	493 (72.7)	185 (27.3)	678 (100.0)	
**The status of employment while studying**				
Working while studying	286 (69.6)	125 (30.4)	411 (100.0)	0.138
Not working while studying	473 (73.9)	167 (26.1)	640 (100.0)	
Overall	759 (72.2)	292 (27.8)	1051 (100.0)	

* χ2-test; N—number of participants.

**Table 3:** Participants with or without IA according to the main reason for the usage of the Internet.

The Main Reason for the Usage of the Internet	N (%)			*p* *
	Participants with IA	Participants without IA	Overall	
University assignments	153 (52.2)	140 (47.8)	293 (100.0)	<0.001
Social networking	576 (80.0)	144 (20.0)	720 (100.0)	
Online gaming	30 (78.9)	8 (21.1)	38 (100.0)	
Overall	759 (72.2)	292 (27.8)	1051 (100.0)	

* χ2-test; N—number of participants.

**Table 4:** Participants with IA according to the main reason for the usage of the Internet and dominant personality trait.

Dominant Personality Trait	The Main Reason for the Usage of the Internet N (%)				*p* *
	University Assignments	Social Networking	Online Gaming	Overall	
Extraversion	27 (18.8)	109 (75.7)	8 (5.5)	144 (100.0)	0.039
Agreeableness	22 (25.3)	64 (73.6)	1 (1.1)	87 (100.0)	
Conscientiousness	34 (29.8)	77 (67.6)	3 (2.6)	114 (100.0)	
Neuroticism	6 (9.3)	57 (89.1)	1 (1.6)	64 (100.0)	
Openness to new experiences	16 (16.8)	73 (76.9)	6 (6.3)	95 (100.0)	
Balanced personality traits	48 (18.8)	196 (76.9)	11 (4.3)	255 (100.0)	
Overall	153 (20.1)	576 (75.9)	30 (4.0)	759 (100.0)	

* Fisher exact test; N—number of participants.

## Data Availability

The data presented in this study are available on request from the corresponding authors.
